# Transcriptome Analysis of Testis from HFD-Induced Obese Rats (*Rattus norvigicus*) Indicated Predisposition for Male Infertility

**DOI:** 10.3390/ijms21186493

**Published:** 2020-09-05

**Authors:** Ahmed M. El-Shehawi, Samir El-Shazly, Mohamed Ahmed, Mohamed Alkafafy, Samy Sayed, Samy Farouk, Saqer S. Alotaibi, Mona M. Elseehy

**Affiliations:** 1 Department of Biotechnology, Faculty of Science, Taif University, Taif 21974, Saudi Arabia; dr_alkafafy@yahoo.com (M.A.); dmrasamy@yahoo.com (S.F.); saqer@tu.edu.sa (S.S.A.); 2 Department of Genetics, Faculty of Agriculture, University of Alexandria, Alexandria 21526, Egypt; monaahmedma@yahoo.com; 3 Department of Biochemistry, Faculty of Veterinary Medicine, Kafrelsheikh University, Kafr Elsheikh 33511, Egypt; elshazlysamir@yahoo.com; 4 Faculty of Veterinary Medicine, University of Sadat City, Sadat City 32958, Egypt; m_m_ahmed2000@yahoo.com; 5 University College of Ranyah, Taif University, Taif 21974, Saudi Arabia; samy_mahmoud@hotmail.com; 6 Faculty of Agriculture, Cairo University, Giza 12613, Egypt

**Keywords:** obesity, transcriptome analysis, gene expression, spermatogenesis, infertility

## Abstract

Obesity is a worldwide life-threatening metabolic disorder, associated with various chronic diseases, including male infertility. Obesity was induced by high fat diet (HFD), and testis RNA was used for the transcriptome analysis using RNAseq via Illumina NovaSeq 6000 System and NovaSeq 6000 Kit. Gene expression level was estimated as FPKM (Fragments Per Kilobase of transcript per Million mapped reads). Differential expressed genes (DEGs) were annotated against gene ontology (GO) and KEGG databases. More than 63.66 million reads per sample were performed with 100 bp cutoff and 6 Gb sequencing depth. Results of this study revealed that 267 GO terms (245 biological processes (BP), 14 cellular components (CC), eight molecular functions (MF)), and 89 KEGG pathways were significantly enriched. Moreover, total numbers of 136 genes were differentially expressed (107 upregulated, 29 downregulated) with |FC| ≥ 2 and bh adjusted <0.05. Interesting DEGs were detected, including obesity and lipid metabolism-related genes, immune response-related genes, cytochrome P450 genes, including aromatase were upregulated, whereas genes related to male fertility and fertilization, cell adhesion, and olfactory receptors were downregulated. The combined expression pattern of the DEGs in obese animals indicated an increase in cholesterol metabolism. Furthermore, high aromatase activity enhances the testosterone turnover into estradiol and lowers the testosterone/estradiol (T/E) ratio, which ultimately reduces fertility. In addition, downregulation of cadherens junction components genes leads to the pre-mature release of sperm from Sertoli cells resulting in the reduction of fertility. Moreover, the downregulation of olfactory receptor genes reduces the chemotaxis capacity of sperms in tracking the oocyte for fertilization, which reduces male fertility. Furthermore, various obesity molecular markers were detected in our transcriptome. The results of this study will enhance our understanding of the molecular network of obesity development, development of obesity novel molecular diagnosis markers, molecular bases of obesity-induced infertility, and the development of anti-obesity drugs.

## 1. Introduction

Obesity is one of the modern health risks worldwide with increasing prevalence in all societies. It has been substantially increased over the last four decades [1,2]. Obesity has been tripled since 1957. In 2016 about 39% (1.9 billion) of the world population aged 18 years and older were overweight, and 13% (650 million) were obese from the same age group [3]. It is expected that the global obesity incidence could level to 18% in men by 2025, and more than 22% in women [4]. Obesity is a health condition resulting from a combined metabolic dysregulation, due to a higher energy input/expenditure ratio [1]. Importantly, obesity is considered a vital predisposing risk factor for various chronic diseases, such as type-2 diabetes, insulin resistance, hypertension, dyslipidemia, chronic inflammation, cancer, cardiovascular diseases, and infertility [5,6,7,8]. Currently, these obesity-associated metabolic disorders are involved in increased mortality and morbidity rates [5]. Moreover, it is well established that there are several important signaling pathways linking obesity to the immune system [9].

Obesity and its various associated metabolic disorders are implicated in reduced male fertility at the level of spermatogenesis, sperm quality, and fertilization capacity [8,10,11]. Epidemiological studies revealed a correlation between obesity and reproductive disorders [12]. Accordingly, azoospermia and oligospermia were more common in obese males, than in non-obese ones [13]. In addition, male obesity is progressively associated with a reduction in sperm quality especially altering the physical and molecular structure of the testicular germ cells and mature sperms [14] reduction of sperm motility and testosterone level while increasing of sperms abnormalities [5,15]. Similarly, it was reported that obesity has contributed to a significant decrease in the count of testicular spermatids and spermatozoa, and a significant decrease in the average daily sperm production. In addition, the acceleration of sperm transportation time can affect sperm maturation, which reduces the sperm quality in rats. Sperm mobility in rats also tends to be associated with a decreased percentage of sperm possessing progressive motility [16,17].

Hyperinsulinemia and hyperglycemia are very common in obese individuals and are consistent key factors in many male obesity studies in rodents [18]. In the meantime, hyperinsulinemia and hyperglycemia have been shown to have an adverse impact on sperm quantity and quality, and may, thus, be related to the decreased fertility seen in obese men [19]. The integrity of sperm DNA is indispensable for successful fertilization and normal embryonic growth [20]. Several human and animal studies have shown that, in spite of different methodologies used to measure sperm DNA integrity, there is an association between obesity and decreased sperm DNA soundness [5,21]. White adipose tissue is responsible for the production of aromatase, adipose-derived hormones, and adipokines that are elevated in obese men [22]. Aromatase, a cytochrome P450 enzyme (CYP19A1), is synthesized in many tissues, including adipose tissue and the testicular interstitial cells of Leydig. It transforms testosterone into estrogens in men [23]. Another main hormone that is synthesized by the white adipose tissue is leptin, which is a key player in controlling energy intake, expenditure, and body weight [24,25]. Moreover, increased levels of leptin greatly reduced testosterone production from Leydig cells [24]. Parallel to the obesity rates, infertility levels have risen [26]. Monitoring the total sperm count and sperm motility in males revealed an annual decline of 1.5% in certain countries with high obesity incidence [27]. Increasing evidence indicates that obesity affects reproductive health in men and induces late-onset male hypogonadism [28], with low serum testosterone levels and associated symptoms [29,30].

The review of the transcriptional response of a set of marker genes in the testis by quantitative real-time PCR showed mild, but substantial, upregulation of the CYP2E1, CYP19A1, TNF and PPAR-y genes in obese mice compared to lean ones, indicating a local response in testicular cells to the HFD regimen with a possible effect on spermatogenesis [31]. Obese mice fed on HFD displayed higher rates of mRNA expression of TNF-α, MCP-1, and F4/80 in the testis, whereas extended administration reported an improvement in sperm quality by reducing the expression of pro-inflammatory cytokines [32]. In addition, in vitro studies have shown how inflammatory cytokines, such as TNFα, IL-1, IL-6, synthesized by white adipocytes can directly affect spermatogenesis through various mechanisms: Alteration of the blood-testis barrier by impairing gap junctional communication in Sertoli cells [33], decreasing spermatogonia cell differentiation, inhibition of meiotic DNA synthesis, and decline of sperm motility [32].

The effect of obesity on gene expression levels was conducted at several genes. Moreover, although obesity has been correlated with several pathological conditions, the evidence of correlating the reduced fertility in males with obesity is still conflicting. Thus, the current study aimed to evaluate the interplay between obesity and male fertility by analyzing testicular transcriptome in obese rats compared to the normal.

## 2. Results

In this study, more than 6.5 billion bases were read in the raw data ranging from 6.5 (C2) to 8.1 (O2) billion bases across samples, whereas above 6.4 billion bases in the trimmed data ranging from 6.4 (C2) to 8.0 (O2). Total reads ranged from 64.41 (C2) to 80.27 (O2) millions in the raw data and ranged from 63.66 (C2) to 79.64 (O2) millions after trimming. The read base quality was above 98.53 (Q20, phred quality of 20) and 95.41 (Q30) in the raw data, whereas it above 98.76 (Q20) and 96.30 (Q30) after trimming (Table S1). 

### 2.1. Quality of Obtained Data

#### 2.1.1. Distribution of Detected Genes Counts 

Distribution of genes, according to their counts, is an important indication of transcriptome analysis quality. In this study, a total number of detected genes was 17,302, among them 4272 genes were excluded because they had at least one read with zero count during sequencing. The remaining 13,030 genes with zero counts were included in the analysis (Figure S1). Exclusion of about 25% of the detected genes substantially validates the dataset used in the further analysis. 

#### 2.1.2. Distribution of Detected Gene Expression Level

Distribution of gene expression level of expressed gene in transcriptome analysis is an important indicator of data set quality. Distribution of expression level in C and O groups is shown in Figure S1. It is clear that the frequency of expression level decreased with the increasing level of expression indicating that more genes had low expression level, compared to those genes with high expression level. This general distribution pattern was noticed in both control and obese groups (Figure S1).

#### 2.1.3. Differentially Expressed Genes (Degs)

The 13030 DEGs were used in the analysis of DEG distribution according to their expression level. Using |FC| ≥ 2 of O/C, the 697 genes were differentially expressed and distributed into two groups. A number of 549 genes were upregulated, and 148 were downregulated. With |FC| ≥ 2 and raw *p*-value < 0.05, 335 DEGS were detected; including 268 upregulated genes and 67 downregulated genes.

**Figure 1:**
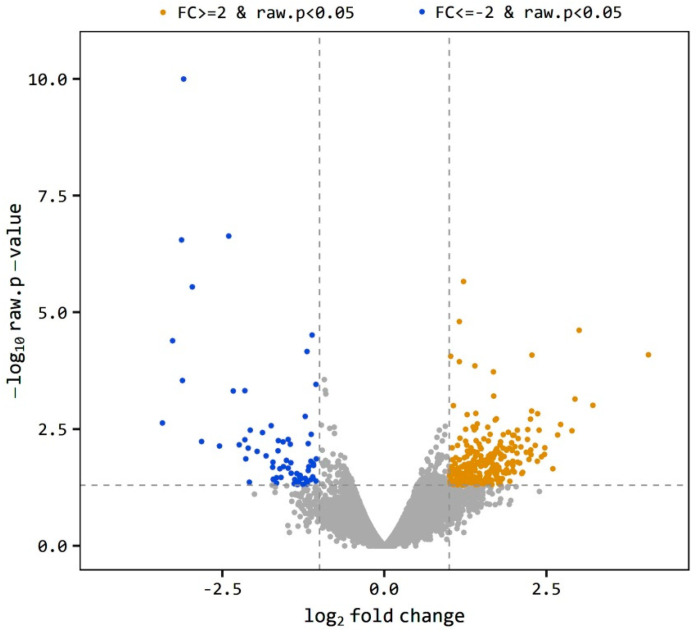
Volcano plot of expression level distribution of differential expressed genes (DEGs) in the testis of obese and control rats with |FC| ≥ 2 (brown dots) and |FC| ≥ 2 (blue dots), *p* < 0.05. The expression level is expressed as O/C.

The level of gene expression was estimated as the ratio of the expression level of a gene in O divided by the expression level of the same gene in the C group indicated as O/C. A gene is considered differentially expressed when O/C equal to or more than 2 or equal to or less than −2 (|FC| ≥ 2). The total 697 DEGs was reduced to 335 genes at *p* < 0.05. The volcano plot (Figure 1) shows the distribution of the DEGs between obese and control samples. The plot is a representation between -log *p*-value and log2 FC. Expression of genes is considered differential when they are located out of the FC limits (|FC| ≥ 2). The closer the distance from the FC baseline, the lower the FC value is. Genes located to the left FC limit are downregulated (blue dots), and those located to the right of the FC limit are upregulated (brown dots). This distribution was noticed in both obese and control samples, yet few genes were highly downregulated (Figure 1). Using a more restricted level of DEGs detection (|FC| ≥ 2, bh-adjusted *p* < 0.05), only 136 DEGs were detected, including 107 upregulated and 29 downregulated genes. This final group of DEGs was used in the functional analysis of DEGs.

#### 2.1.4. Gene Ontology (GO)

**Figure 2:**
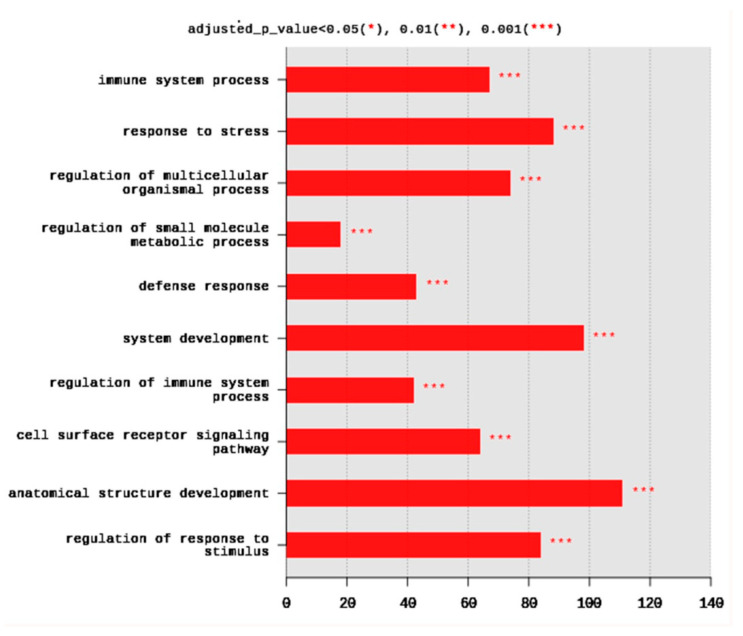
Distribution of the top 10 gene ontology (GO) enriched biological processes (BP) terms, adjusted *p* < 0.05. The Y axis represents the enriched BP terms, X axis represents the intersection size (the number of DEGs for a BP term).

The set of DEGs was used to test their enrichment by annotation on GO database using g:Profiler. The annotation usually detects the overrepresented GO terms. Gene ontology showed that DEGs were enriched in the three major GO groups; biological processes (BP), cellular components (CC), molecular functions (MF). A total of 245 BP, 14 CC, and 8 MF terms were significantly (adjusted *p* < 0.05) enriched. The top 10 BP terms enriched at *p* < 0.001 are shown in Figure 2. They included immune system process (67 DEGs), response to stress (88 DEGs), regulation of multicellular organismal process (74 DEGs), regulation of small molecule metabolic process (18 DEGs), defense response (43 DEGs), system development (98 DEGs), regulation of immune system process (42 DEGs), cell surface receptor signaling pathway (64 DEGs), anatomical structure development (111 DEGs), and regulation of response to stimulus (84 DEGs) (Figure 2).

**Figure 3:**
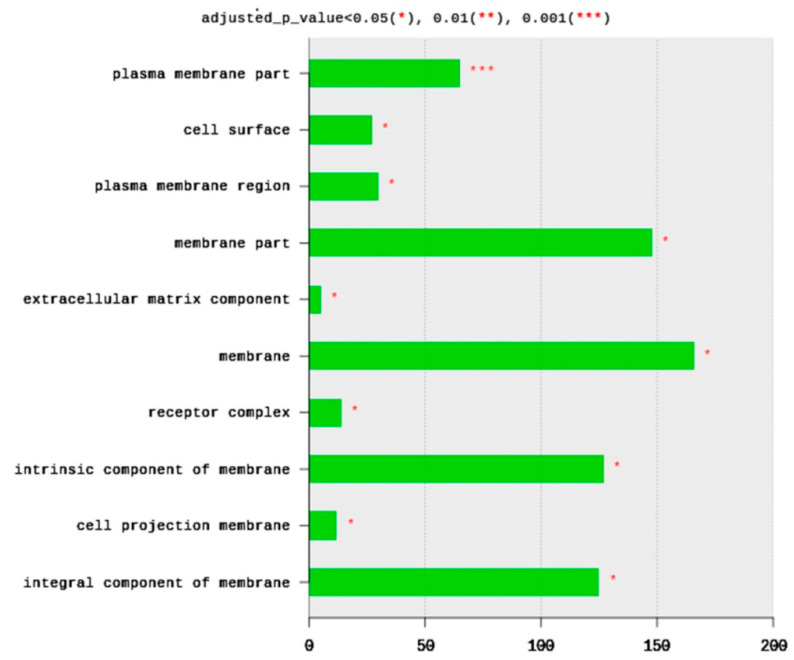
Distribution of GO top 10 enriched cellular components (CC) terms, adjusted *p* < 0.05. The Y axis represents the enriched BP terms, X axis represents the intersection size (the number of DEGs for a CC term).

The total number of CC terms was 283, with 14 significantly (adjusted *p* < 0.05) enriched terms (Figure 3). The top 10 CC terms included plasma membrane parts (65 DEGs), cell surface (27 DEGs), plasma membrane region (30 DEGs), membrane part (148 DEGs), extracellular matric component (five DEGs), membrane (166 DEGs), receptor complex (14 DEGs), an intrinsic component of membrane (127 DEGs), cell projection of membrane (12 DEGs), and integral component of membrane (125 DEGs). All enriched terms belong to plasma membrane structure and function. Based on the significance level (adjusted *p*-value), the most enriched term is the plasma membrane part and cell surface (Figure 3).

**Figure 4:**
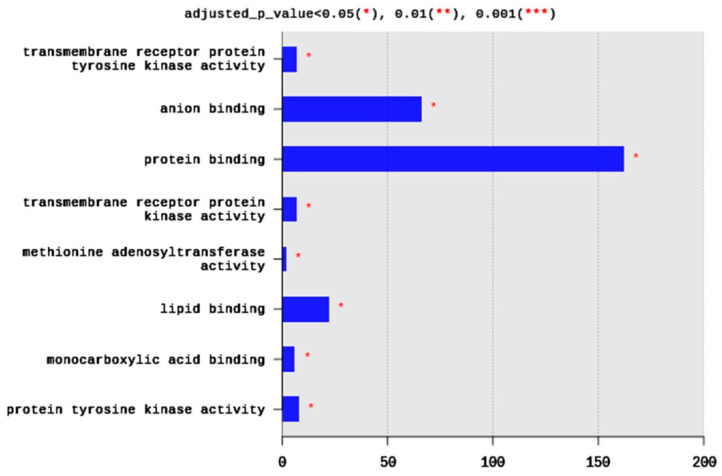
Distribution of GO top 10 enriched molecular functions (MF) terms, adjusted *p* < 0.05. The Y axis represents the enriched BP terms, X axis represents the intersection size (the number of DEGs for an MF term).

Regarding MFs, 285 terms were enriched, yet only eight terms were significantly enriched, including transmembrane receptor protein tyrosine kinase activity (seven DEGs), anion binding (66 DEGs), protein binding (162 DEGs), transmembrane receptor protein kinase activity (seven DEGs), methionine adenosyltransferae activity (two DEGs), lipid binding (22 DEGs), monocarboxylic acid binding (six DEGs), and protein tyrosine kinase activity (eight DEGs) (Figure 4). Generally, the enriched MFs belong to signal transduction pathways and binding activities (Figure 4).

### 2.2. KEGG Analysis

**Figure 5:**
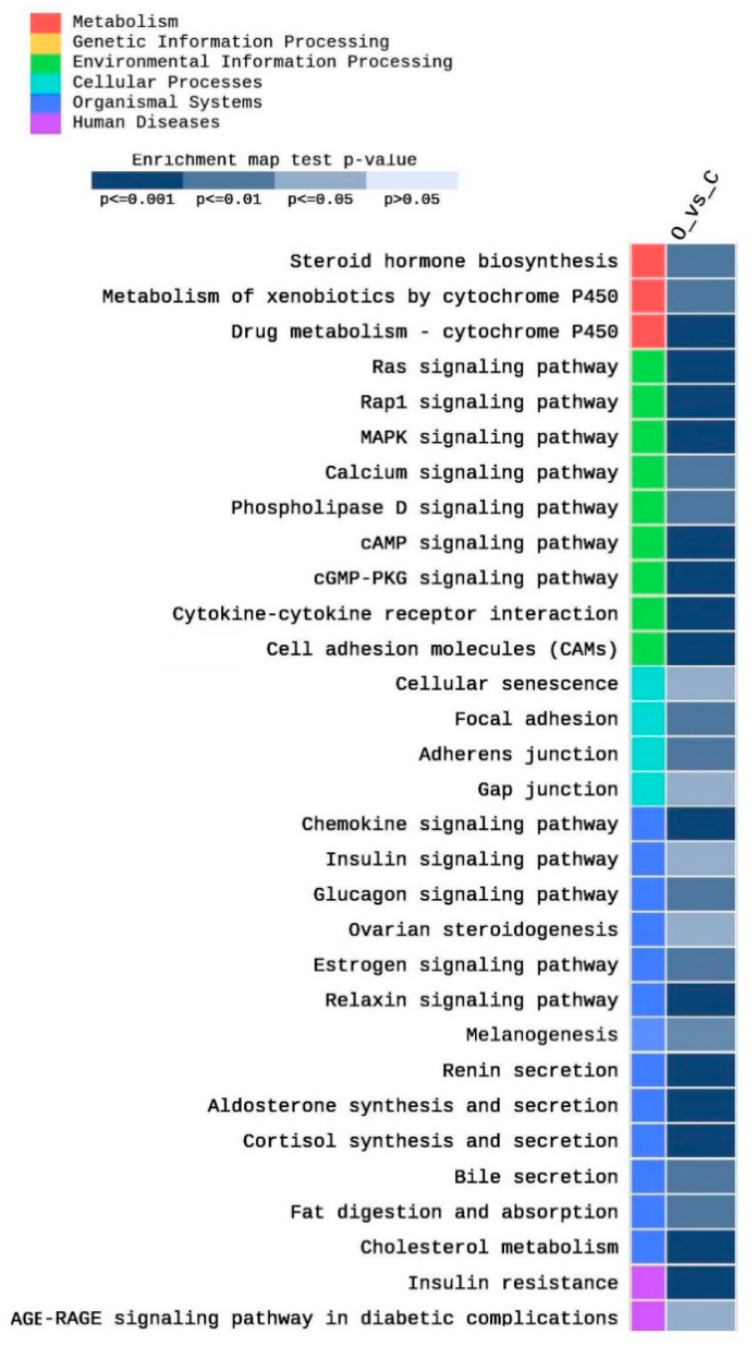
Heat map of the enriched pathways of the KEGG database after filtration of the significantly expressed genes (|FC| > 2) and adjusted *p* < 0.05.

A total of 255 KEGG pathways were enriched in this study as an impact of obesity on testis function, as well as other related metabolic pathways. Among them, 89 pathways were significantly (*p* < 0.05) enriched. Figure 5 shows partial heatmap of the KEGG enriched pathways (adjusted *p* < 0.05, |FC| > 2). They included pathways related to cholesterol metabolism and steroid hormone metabolism, such as cholesterol metabolism (five DEGs), steroid hormone biosynthesis (four DEGs), ovarian steroidogenesis (three DEGs), cortisol synthesis and secretion (five DEGs) (Figure 5). Moreover, different Cytochrome P450 (CYP) pathways, including the metabolism of xenobiotics by cytochrome P450 (four DEGs) drug metabolism–cytochrome P450 (five DEGs) were enriched. Furthermore, various signaling pathways, including Ras (ten DEGs), Rap1 (12 DEGs), cAMP (nine DEGs), cGMP-PKG (seven DEGs), MAPK (nine DEGs), estrogen (five DEGs), relaxin (six DEGs), phospholipase D (five DEGs), AGE-RAGE in diabetic complications (four DEGs), insulin (four DEGs), glucagon (five DEGs), calcium (six DEGs), and chemokine (nine DEGs) signaling pathways were enriched (Figure 5). In addition, several cell adhesion pathways were enriched, including cell adhesion molecules (nine DEGs), focal adhesion (six DEGs), adherens junction (four DEGs), and gap junctions (four DEGs). Moreover, fat digestion and absorption (four DEGs), as well as bile secretion (four DEGs) pathways, were enriched. Other obesity-related pathways were enriched, including insulin resistance (six DEGs), renin secretion (five DEGs), aldosterone synthesis and secretion (six DEGs), and melanogenesis (five DEGs).

### 2.3. Gene Expression

#### 2.3.1. Obesity and Lipid Metabolism

Several obesity- and lipid-metabolism-related genes were differentially expressed in the testis of obese rats, including fatty acid synthesis, such as acyl-CoA thioesterase 4 (O/C −10.75) or fatty acid elongation, including 3-hydroxyacyl-CoA dehydratase 4 (O/C 4.81). Moreover, other DEGs involved in the protection of obesity, such as apolipoprotein C3 (O/C 7.66), phospholipase C beta 1 (O/C −8.55), and phospholipase A2, group IIF (O/C 3) were differentially expressed. In addition, genes involved in steroid biosynthesis (carboxyl ester lipase, O/C −3.9), fatty acid oxidation (acyl-CoA oxidase-like, O/C −4.22), cellular response to lipids (pyruvate dehydrogenase kinase 4, O/C 4.69), and lipid transport and metabolism (low-density lipoprotein receptor, O/C 3.81) were significantly differentially expressed (Table 1). 

#### 2.3.2. Obesity Marker Genes (OM)

DEGs included a wide range of obesity marker genes, including obesity and lipid metabolism genes, such as acyl-CoA thioesterase, carboxyl ester lipase, and apolipoprotein C3. Moreover, two of the detected olfactory receptors serve as Oms; olfactory receptor 434, olfactory receptor 472. In addition, four of the detected CYPs are Oms. Furthermore, protocadherin gamma subfamily B, 7 and all immune response genes detected in this study are Oms (Table 1).

#### 2.3.3. Spermatogenesis, Reproduction, Fertilization

Five genes related to spermatogenesis, sperm maturation, and fertilization were differentially expressed in the testis of obese rats. This group included spermatogenesis associated 13, spermatogenesis associated serine-rich 2-like, sperm associated antigen 11b-like, estrogen-related receptor gamma, anterior gradient 2, protein disulphide isomerase family member with an O/C 7.42, 2.9, −3.18, −9.63, and −7.8 consecutively (Table 1). 

#### 2.3.4. Cytochrome P450 (CYPs)

DEGs included five different CYPs, four CYPs were upregulated; CYP2a1, CYP4f1, CYP7b1, CYP19a1 (aromatase), whereas one; CYP2d3 was downregulated (Table 1).

#### 2.3.5. Cell Adhesion

Three cell adhesion genes were differentially expressed, including protocadherin gamma subfamily B, 7 that was downregulated with an O/C −2.96, carcinoembryonic antigen-related cell adhesion molecule 9 that was downregulated with an O/C −2.74, and carcinoembryonic antigen-related cell adhesion molecule 1 which was upregulated with an O/C 3.07, (Table 1).

#### 2.3.6. Immune Response

Seven immune response genes were upregulated in the current study, including C-C motif chemokine ligands 24, C-C motif chemokine ligands 6, C-X-C motif chemokine ligand 9, interleukin 22 receptor subunit alpha 1, interleukin 17 receptor E, and Cd4 molecule with an O/C of 3.57, 2.75, 2, 5.53, 3.3, 6.37, consecutively (Table 1).

#### 2.3.7. Olfaction

Three olfactory receptors were detected with significant downregulated expression profile, including olfactory receptor 1710 (O/C −8.68), olfactory receptor 434 (O/C −7.07), and olfactory receptor 472 (O/C −4.23) (Table 1).

## 3. Discussion

Transcriptome analysis of testis from obese rats revealed interesting, unique features compared to the control rats. First, various GO groups were enriched, including BPs, CCs, and MFs. A number of 136 genes were significantly differentially expressed (107 upregulated, 29 downregulated). These results indicated the high impact of obesity at the gene expression level. It is noteworthy that obesity-induced by HFD in this study was due to a significant increase in body weight, body mass index (BMI), body fat content, liver fat deposition, and higher blood lipids [34]. Various GO terms were significantly enriched in the current study with various numbers of DEGs, suggesting the multidimensional impact of obesity not only on body weight, but also on internal stress on the biological system of obese individuals.

Obesity is the result of a disruption of energy balance that leads to weight gain and metabolic disturbances, which causes tissue stress and dysfunction [35]. However, recent findings have highlighted the overall negative impact of obesity on immunity and pathogen defense [36]. Moreover, the detrimental effects of obesity on immunity are associated with alterations in lymphoid tissue architecture and integrity and shifts in leukocyte populations and inflammatory phenotypes [37]. In consistence with previous results, the predominant enriched BP terms were related to immune response (immune system, regulation of immune system process), response to stress (response to stress, defense response, and regulation of response to stimulus), and cell surface receptor signaling pathways.

Since changes in the dynamic properties of the cell membrane could be one of the events by which obesity affects insulin sensitivity, the importance of cell membrane lipids as essential regulators of insulin resistance has been considered [38]. It is noteworthy that obesity extensively targets cell surface components, including membrane structure, integral membrane part, cell projections, and membrane receptors. Recently, it was confirmed that metabolic syndrome, such as insulin resistance, hypertension, obesity, and hyperglycemia are associated with alterations in membrane lipids and modified lipid composition in the plasma membrane, as well as high risk of type-2 diabetes and cardiovascular disease [39]. Interestingly, most of the significant CC terms were related to membrane structure (plasma membrane parts, cell surface, extracellular matrix component, receptor complex, an intrinsic component of membrane, cell projection of membrane, and integral component of the membrane).

Various GO and MF terms detected in this study were related to protein kinase activities. This is in agreement with previously reported data, which indicated that obesity-induced inflammation, repress insulin receptor signaling (IRS), and contribute to insulin resistance development. Recently, it was reported that obesity was associated with the regulation of various stress kinases activity, such as mitogen-activated protein kinase (MAPK), c-Jun NH2-terminal kinase (JNK), an inhibitor of NF-kB kinase complex beta (IKKβ), AMP-activated protein kinase (AMPK), protein kinase C (PKC), Rho associated coiled-coil containing protein kinase (ROCK) and RNA-activated protein kinase (PKR) [40].

### 3.1. KEGG Pathways

Concerning KEGG pathways, more than 89 KEGG pathways were significantly enriched in the testis of obese rats compared to the control ones. Previous studies reported an association of these pathways to obesity in the testis or other tissues of obese specimens. For example, the mitogen-activated protein kinase (MAPK) pathway activates protein kinases that are involved in cell proliferation, differentiation, and cell death. They are activated through G-protein coupled receptor (GPCR). JNK subfamily of MAPK is induced by abiotic stresses, as well as inflammatory cytokines produced in obese individuals [41]. MAPK signaling pathway is involved in spermatogenesis, sperm maturation, capacitation, and acrosome reaction [42]. It also modulates cell adhesion through the regulation of adherens junction via RapI pathway [43]. This gives a reasonable explanation for the enrichment of MAPK in the testis of obese rats in the current study. Enrichment of Phospholipase D (PLD) pathway supports its previously reported protective role from obesity. Mice deficient in PLD1 and PLD2 consumed more food and developed obesity, as well as resistance indicating PLD protective role against obesity and its associated metabolic disorders [44]. cAMP, cGMP pathways were enriched because they are linked to the induction of several other enriched pathways in this study. For example, cAMP pathway is involved in the induction of Rap1, Ras, glucagon, and calcium signaling pathways [45,46], whereas cGMP is involved in the invoking of relaxin and MAPK signaling pathways [47]. Furthermore, cGMP, cAMP, and calcium signaling pathways play an essential role in olfactory receptors functions [48]. 

Ovarian steroidogesis, as well as cortisol synthesis and secretion pathways, were highly enriched. These two pathways use cholesterol to produce estrogen cortisol, consequently, which causes depletion of cholesterol reservoir destined for testosterone biosynthesis in the testis. This will result in a lower Testosterone/Estrogen (T/E) ratio, and consequently, lower fertility in the obese male rats compared to the control group. It was previously reported that obesity increases the biosynthesis of cortisol from cholesterol [49]. Similar results were obtained in mice where HFD decreased testosterone level, increased estradiol level, and consequently, decreased sperm motility compared to the control indicating impaired fertility [50]. Relaxin play an indispensable role in the regulation of appetite and body weight. Previous data showed an association between relaxin, obesity, and diabetes, suggesting a role for the relaxin/insulin pathway in the development of metabolic disorders, including obesity [51]. Ectopic expression of melanogenesis-related genes was reported in adipose tissue in obese humans, indicating an anti-inflammatory and antioxidant function of melamine in the adipose tissue as a result of obesity [52]. High levels of aldosterone and renin were reported in adipose tissue in obese individuals [53]. Because obesity increases sodium retention, which results in salt-sensitive hypertension, it induces higher levels of aldosterone and renin by invoking the aldosterone-renin–angiotensin system [53]. In obese mice, during insulin resistance, glucagon is increased and results in the release of higher levels of calcium, which induces calcium signaling leading to induction of higher insulin signaling [54]. AGE/RAGE signaling is involved in different diseases, especially diabetes. It functions mainly through PKC, p38 MAPK, and ERK1/2 signaling pathways. It was also reported that it increases oxidative stress during diabetes and decrease SOD-1 expression [55].

### 3.2. Gene Expression Analysis

#### 3.2.1. Lipid Metabolism and Control of Obesity

Seven DEGs were categorized under the pathway of lipid metabolic processes. Acyl-CoA thioesterases (Acots) are a group of enzymes that catalyze the hydrolysis of fatty acyl-CoA thioesters to the corresponding free fatty acids and coenzyme A [56]. In the present study, Acyl-CoA thioesterase 4(ACOT4) and Acyl-coA oxidase-like (ACOXL) expression were downregulated in the testis of obese rats with an O/C value of −10.75 and −4.72, respectively. ACOT4 mRNA expression was found to be downregulated in the gonadal white adipose tissue when high fat diet was used compared with fasting animals [57]. The regulation of the entry of glycolytic products into the tricarboxylic acid cycle is catalyzed by Pyruvate dehydrogenase complex (PDC). The activity of the PDC is upregulated and downregulated by pyruvate dehydrogenase kinase and pyruvate dehydrogenase phosphatase, respectively [58]. The obtained results showed that PDK4 was highly expressed in the testis of obese rats with an O/C of 4.69 value. Previous studies have shown that a short-term high fat diet increases PDK4 protein abundance [59]. Another enzyme, carboxyl ester lipase (CEL) plays a major role in the hydrolysis, and absorption of lipids, was found to be downregulated with an O/C of −3.9. In consistence with our results, it was observed that high fat diet (HFD) supplementation decreased mRNA level of CEL [60], and its knockout led to the protection of diet-induced obesity [61]. ApolipoproteinC3 (APOC3) is lipoprotein lipase (LPL) inhibitor that induces obesity and develops insulin resistance [62]. Its expression was upregulated in obese rats with an O/C 7.66 compared to the control rats. Mice deficient in APOC3 showed an improvement in fatty acid uptake from triglycerides plasma of adipose tissue. This ultimately led to a higher risk of diet-induced obesity and the development of insulin resistance [63]. As serum triacylglycerol level was elevated in obese rats, a strong positive correlation between plasma apoCIII and TG concentrations has been invariably observed in human and animal studies [64]. Low-density lipoprotein receptor (LDLr) function requires the binding of apolipoprotein B (ApoB)- and ApoE-enriched cholesterol particles with the subsequent endocytosis of the low-density lipoprotein (LDL) receptor and uptake of the cholesterol-containing lipoprotein particles into the cell [65]. LDLr was highly expressed in the current study (O/C 3.81) in agreement with previously reported results where spermatozoa of obese patients showed more intense positive staining for LDLr [66]. Diaacylglycerol kinases (DGKs) catalyze the reaction that removes DAG, by its conversion to phosphatidic acid at the plasma membrane, endoplasmic reticulum, and the nucleus, and thereby terminates DAG-derived signals [67]. We found that the enzyme was upregulated in obese mice with an O/C of 2.35. Mice deficient in the DGKƹ fed on HFD showed adipocyte fat accumulation and insulin resistance [68]. Overexpression of DGKƹ in obese rats in this study is consistent with these findings in that DGKƹ is expected to protect animals from obesity. Phospholipase C is a crucial enzyme for the phosphoinositol pathway and whose activity is involved in eukaryotic signal transduction as it generates two second messengers: Diacylglycerol (DAG) and inositol 1,4,5-trisphosphate (IP3) [69]. Phospholipase C B1 (Plcb1) was drastically reduced with an O/C of −8.55. Increased phospholipid content was observed in aged testicular tissues, which indicated a negative impact on spermatogenesis and male fertility [70].

#### 3.2.2. Spermatogenesis and Fertilization

In this study, several genes in different categories were found to be associated with fertility. Testosterone biosynthesis in the Leydig cells is controlled by the action of two steroidogenic proteins; steroidogenic acute regulatory protein (STAR) and cytochrome P450 17a-hydroxylase (P450c17) [71]. The expression of these two proteins is controlled by the estrogen-related receptor gamma (ERRg). Overexpression of ERRg in mouse Leydig cells led to elevated P450c17 expression at mRNA, as well as protein levels, and consequently, higher testosterone levels. This indicates that ERRg regulates the testicular steroidogenesis directly by inhibition of P450c17 or indirectly by suppression of cAMP induction of STAR [71]. Expression of ERRg was highly suppressed in the testis of obese rats with an O/C ratio of −9.63—giving the second highly repressed gene in this study. Repression of ERRg in obese testis will ultimately decrease the level of testosterone production, which is involved in sperm maturation, and consequently, reduction of fertility compared to the control.

Sperm associated antigen 11b, another downregulated gene, (SPAG11B) was severely downregulated with an O/C value of −30.17. This gene encodes various epididymis-specific secretory proteins, which are androgen-dependent. It is suggested that these proteins are involved in sperm maturation. They have similar protein domains to beta-defensins antimicrobial peptides [72]. Protein disulphide isomerase (PDI) was highly downregulated with an O/C of −7.8, indicating that obesity not only affects the production of sperms, but also their maturation and their capability of fertilization. Different inhibitors of protein disulfide isomerase (PDI) activity were able to inhibit sperm-egg fusion in vitro [73]. PDIs were associated with epididymal sperm maturation and could be attractive candidates for monitoring male fertility [74]. It was also demonstrated recently that the amount of PDIA3 is reduced in the spermatozoa of male individuals with obesity-associated asthenozoospermia [75]. In line with our results, a recent study showed that although switching from HFD to the normal diet reduced body weight and fat content—it caused irreversible changes in testicular metabolism and sperm quality, including sperm viability, pyruvate and glutamate metabolism, ammonia recycling, urea cycle, and glutathione content [76]. 

On the contrary, the expression of two genes was upregulated, including spermatogenesis associated serine-rich 2-like (SPATS2L) and spermatogenesis associated 13 (SPATA13). SPATS2L was highly expressed in obese rats compared to lean ones with an O/C ration of 2.9. It showed high expression in the gall bladder (RPKM 14.3) and other 26 tissues, including testis [77]. Meanwhile, SPATA13 was highly expressed in the testis with an O/C value of 7.42. It is a guanine nucleotide exchange factor (GEF) found in specific regions of the adult brain that control anxiety, fear and threat, pain, feeding, addiction, voluntary activity, and aggressive behavior [78]. 

#### 3.2.3. Cell Adhesion and Fertility

The interconnection among BMI, steroidogenesis, spermatogenesis, and male infertility have been elaborately studied [14]. Numerous studies have corroborated these findings to strongly suggest the disrupting impact of obesity upon male fertility [79,80]. 

Cadherins are the main components of adherins junctions (Ajs) responsible for inter-Sertoli and Sertoli–germ cell adhesion [81,82] and the structure of desmosomes in the testis [82,83]. Disruption of adherens junction, due to the loss of vascular endothelial (VE) cadherein caused the premature release of spermatids from Sertoli, which increases infertility [43,84]. This confirms that cadherins are involved in all stages of sperm maturation and release [85]. Protocadherin gamma B7 was significantly downregulated with an O/C −2.96. Moreover, several cell adhesion pathways were enriched in the KEGG database, including cadherens junction. Downregulation of protocadherin agrees with previously reported data, and suggests that obesity reduces fertility via the premature release of spermatids from Sertoli cells, due to the downregulation of cadherins. This agrees with results obtained by Fan et al. 2015, where HFD decreased the adhesion of spermatogenic cells to Sertoli cells and disrupted the testis blood barrier [50].

Carcinoembryonic antigen-related cell adhesion molecule (CEACAM) is produced from the liver and is involved in the development of insulin resistance and obesity. The reduction of hepatic CEACAM1 caused insulin resistance and obesity in mice and other species [86]. In the present study, two carcinoembryonic antigen-related cell adhesion molecule genes were differentially expressed in the testis of obese rats. CEACAM1 was upregulated (O/C 3), while CEACAM9 was downregulated (−2.74). Expression CEACAM9 is in line with results reported by Heinrich et al. [86], whereas upregulation of CEACAM1 in the testis contradicts previously reported data [86]. CEACAM9 has not been reported in previous studies and could be testis-specific and has the similar reported role of CEACAM1 in hepatocytes [86]. Overexpression of other CEACAMs (CEACAM1, 5, 6) was a predisposition factor for pancreatic cancer [87].

#### 3.2.4. Olfaction and Olfactory Receptors

Olfactory receptors (Ors) have been thought to be expressed in a tissue-specific manner in the olfactory organ’s epithelial cells [88]. Other studies proved that Ors were expressed in other human organs, including the tongue [89] and prostate [90]. Recently, a comprehensive expression analysis of 387 OR genes in human spermatozoa using RAN-seq led to the detection of 91 transcripts of OR genes [91]. In the current study, three olfactory receptors were significantly downregulated; olfactory receptor 1710 (O/C −8.68), olfactory receptor 434 (O/C −7.07), olfactory receptor 472 (O/C −4.23). Various investigations reported the expression of OR in the testis [92,93,94], and their results suggested that several Ors are involved in sperm chemotaxis in mammals to follow the chemoattractants secreted by the oocyte for ultimately efficient fertilization [93]. Human hOR17–4 (OR1D2) [93] and mouse MOR267-13 [95] were among the early characterized Ors. Therefore, Ors downregulation in the testis of obese rats could decrease sperm chemotaxis, and consequently, fertilization efficiency compared to the control. Several other studies reported similar supporting data where obesity caused the loss of olfactory sensory neurons, disruption in neuronal proliferation, and reduced olfactory discrimination in rats feed on HFD [96]. In addition, gene expression of the odorant receptor co-receptor (DmOrco) in olfactory receptor neurons was reduced significantly by about 70% and 47% in flies fed on HFD for 7 and 14 days, respectively, compared to the flies feed on a normal diet [97].

#### 3.2.5. CYPs expression 

CYP proteins are vital in chemical (xenobiotics) detoxification and various drug metabolism [98,99]. They contribute to the production and metabolism of cholesterol and its derivative steroid hormones. For example, aromatase (CYP19A1) converts testosterone to estradiol (estrogen), which is required for sperm maturation, capacitation, acrosome reaction, and fertilization [100]. Five CYPs enzymes were detected in the transcriptome of testis of obese rats. CYP2D3 was downregulated with an O/C −2.3, where the other four CYP2A1, CYP4F1, CYP7B1, CYP19A1 were upregulated with an O/C 2.51, 2.75, 3.49, 2.1, respectively.

Interestingly, CYP19A1 (aromatase) was upregulated with an O/C 2.1—suggesting an increase of testosterone conversion to estradiol changing the normal ratio of testosterone/estradiol (T/E) required for spermatogenesis and sperm maturation. Higher levels of testicular estradiol impair spermatogenesis and have a negative impact on fertility [100]. Abnormal T/E ratio (T/E <10) was associated with lower sperm parameters and interestingly, aromatase inhibitors reversed this effect and improved sperm quality and fertility [101].

In the rat, CYP2A1 is highly involved in 7-alpha-hydroxylation of testosterone, progesterone, and rostenedione to produce bile acids. It is expressed specifically in the liver and testis [102]. Its high expression will deplete the testosterone level, and consequently, estradiol in the testis, especially with the upregulation of aromatase. CYP2D3 and CYP4F1 are involved in the NADPH-dependent electron transport pathway. They oxidize various compounds, including steroids, into bile acids in the liver [103,104]. This also will consequently decrease the testosterone and estradiol in the testis. CYP7B1, also called oxysterol 7-alpha-hydroxylase, is expressed in the liver and brain, as well as many other tissues, including testis (the human protein atlas, https://www.proteinatlas.org/ENSG00000172817-CYP7B1/tissue). In the liver, the enzyme breaks down cholesterol to chenodeoxycholic bile acid, which is involved in fat digestion. In the brain, it converts cholesterol to neurosteroids, which regulate communication between nerve cells [105]. Its function in the tests is unknown, yet its impact could be speculated using its analogous role in the liver and brain to turn over cholesterol to other cholesterol derivatives in the testis. 

Altogether, the detected expression pattern of the five detected CYPs in this study could lead to the use of cholesterol and other steroids to produce bile acid for fat digestion by liver, lower production of testosterone, higher production of estradiol, and lower T/E ratio in the testis. This condition ultimately could negatively impact spermatogenesis, sperm maturation, and reduces fertility. This is strongly supported by the significant enrichment of cholesterol metabolism to probably unknown cholesterol derivatives in the testis. Obese rats used in this study showed significantly higher body weight, body mass index, body fat accumulation, liver fat deposition, and blood lipids level [35].

#### 3.2.6. Immune Response

Cytokine and chemokine families are extracellular molecular regulators that mediate both immune cell recruitment and complex intracellular signaling control mechanisms that characterize inflammation [106]. C-C motif chemokine and their ligand are vital in insulin resistance development [107,108]. In the present study, three C-C motif chemokine ligand genes were significantly expressed in the testis of obese rats. It seems that these C-C chemokine ligands 6, 9, 24 expressed in this study are testis-specific.

Obesity was proved to induce inflammatory cytokines expression [109]. TNFα and IL-6 are among the major obesity-induced pro-inflammatory cytokines that inhibit insulin signaling and cause insulin resistance [110], whereas IL-10 is a classical anti-inflammatory cytokine which is thought to counter pro-inflammatory cytokine and improve the obesity-induced inflammatory effect, and consequently, insulin resistance [111].

IL-17 and IL-22 are leukocyte-derived cytokines that primarily impact epithelial cells in tissues [112,113]. The pro-inflammatory cytokine interleukin (IL)-17 has been associated with the induction of tissue inflammation. Obese individuals exhibit many symptoms of chronic low-grade inflammation, suggesting that IL-17 may impact adipose tissue [6], while IL-22 is produced by several populations of immune cells at the site of inflammation [114]. In an early study, injection of mice with adenovirus expressing IL-22 induced marked body weight loss in lean mice [115]. The interleukin 17 receptor E and interleukin 22 receptor subunit alpha were highly expressed in the current study, which may indicate a higher level of IL-17, due to obesity and a high level of IL-22 for protection from obesity. 

CD4 is a cell surface glycoprotein produced by CD4 cells, which contributes to the development of obesity-induced inflammation, and consequently, insulin resistance in mice [116]. CD4 T cells producing the CD4 molecule was proved to play a positive role not only in obesity and insulin resistance development, but also in the obesity memory of previous obesity, since their depletion resulted in the loss of this memory [117].

## 4. Materials and Methods

### 4.1. Animals and Experimental Design

Twelve adult male Wistar rats (seven weeks old) were purchased from an experimental animal center, Faculty of Pharmacy, King Abdul-Aziz University, Saudi Arabia. The rats were kept under controlled conditions of temperature (23 ± 2 °C), humidity (50 ± 5%) and 12-h light/dark cycle. Rats were kept for one-week acclimatization before the onset of the experiment. Animals were housed in sanitized polypropylene cages with sterile husk as bedding with free access to standard basal diet and water ad libitum. All procedures were approved by the Animal Care Committee of Taif University (#1-440-6145). After one-week of acclimation, rats were randomly assigned into two groups of 6 each. The control group was kept on standard basal diet ad libitum. HFD group was fed on HFD (1 kg ration contained 164 g casein, 303.1 g corn starch, 90 g dextrin, 115 g sucrose, 58.9 g cellulose, 190 g butter oil, 10 g soya bean oil, 2.1 cysteine, 2.9 g choline tartrate, 35 g mineral mix, and 11.7 g vitamin mix) [35,118]. Animals of this group were fed HFD ad libitum and gained free access to water for eight weeks to induce obesity that was confirmed by elevated serum triacylglycerol level and high BMI. 

### 4.2. Sampling

After induction of obesity had been confirmed, rats were fasted for 12 h and were anesthetized using a mixture of (50 mg ketamine + 5 mg Xylazine)/kg, i.p. Rats were euthanized by head decapitation; testicular tissue samples were immersed immediately in liquid nitrogen and were preserved in −80 Ċ for analysis of gene expression.

### 4.3. RNA Isolation and cDNA Library Construction

Total RNA was isolated using Qiazol reagent following manufacturer procedures (Qiagen, Hilden, Germany). TruSeq Stranded mRNA LT Sample Prep Kit (Illumina, San Diego, CA, USA) was used to purify mRNA and prepare cDNA libraries. Poly-A mRNA was purified using poly-T oligo attached magnetic beads. This was carried out in two stages. During the second stage, mRNA was fragmented to small fragments under high temperatures. Synthesis of cDNA was reverse transcribed from fragmented RNA using reverse transcriptase and random primers. Actinomycin D was included during the first strand synthesis to improve strand specificity by allowing only RNA-dependent synthesis. cDNA was adenylated with a single A at the 3′ end to prevent self-ligation and to allow the adapters with corresponding 3′ T overhang to bind specifically to both ends of cDNA. Adapters-indexed cDNA fragments were enriched with were enriched using PCR and adapters-specific primer cocktail in a 15-cycle PCR reaction. The PCR products were purified using AMPure XP beads. The quality of cDNA libraries was estimated at 2100 Bioanalyzer using a DNA Agilent DNA-1000 chip (Agilent Technologies, Santa Clara, CA, USA). Fragments with insert size between 200–400 bp (260 bp) were selected for paired-end sequencing. Libraries were normalized to 10 nM, and equal volumes were pooled.

### 4.4. Transcriptome Sequencing and Assembly

Clustering and sequencing of cDNA libraries were carried out on the NovaSeq 6000 System and NovaSeq 6000 Reagent Kit (Illumina, San Diego, CA, USA). The sequencing protocol was programed to generate 60 × 10^6^ PE reads of 100 bp with a sequencing depth of 6 Gb. Sequence reads were trimmed by eliminating low quality reads, contaminant DNA, adaptor sequence, and PCR duplicates using Trimmomatic program 0.38 [119]. Using a sliding window method, bases of reads that did not qualify for window size 4 and mean quality 15 were trimmed. Furthermore, it read less than 36 bp were excluded. Quality of sequencing data was checked and combined in one FASTQ file using FastQC v0.11.7 (http://www.bioinformatics.babraham.ac.uk/projects/fastqc). Trimmed reads were mapped to the rat (*Rattus norvegicus*) reference genome UCSCrn6 using HISAT2 version 2.1.0 [120] and Bowtie2 2.3.4.1 (https://ccb.jhu.edu/software/hisat2/index.shtml). StringTie version 1.3.4d [121,122] was used to assemble the uniquely mapped sequences into transcripts (genes). 

### 4.5. Transcriptome Analysis

Gene expression from different libraries was normalized using Trimmed Mean of M-values (TMM) method of edgeR. The abundance of assembled transcripts was calculated as read-count, normalized FPKM (Fragments Per Kilobase of transcript per Million mapped reads), RPKM (Reads Per Kilobase per Million mapped reads), or TPM (Transcripts Per Million) per sample. Gene ontology (GO) analysis was conducted using g:Profiler tool (https://biit.cs.ut.ee/gprofiler) [123] against gene ontology (http://geneontology.org/) database to detect the over-represented gene ontology terms. Usually, it annotates over-/down- expressed genes into three groups; biological processes (BP), molecular functions (MF), and cellular components (CC). KEGG analysis was performed against the KEGG database. KEGG enrichment was conducted on the KEGG pathway homepage (http://www.kegg.jp/kegg/pathway.html) to identify the change in gene expression level in a set of genes and develop the heatmap for expression level. Statistical analysis was performed using EdgeR package of R Version 3.0 [124]. DEGs were detected using the edgeR package based on |FC|≥2 and Benjamini-Hochberg (bh) adjusted *p*-value < 0.05. Enrichment of GO and KEGG pathways was based on the FDR adjusted *p*-value (< 0.05).

## 5. Conclusions

The results of the current study conclude that obesity negatively impacts male sterility through various routs. It causes the depletion of testosterone by activation of cholesterol metabolism for producing bile acids in the liver to meet the excessive fat digestion and high conversion of testosterone to estradiol in the testis. This causes a lower T/E ratio and a reduction of fertility. Moreover, obesity could cause a reduction of sperm chemotaxis through the downregulation of olfactory receptors, which leads to lower fertility. In addition, obesity lowers fertility through reducing the expression of adherenes junction components, which result in the premature release of sperms from Sertoli cell. Results will enhance our understanding of the molecular impact of obesity on male fertility and the development of novel obesity molecular markers and drugs.

## Figures and Tables

**Table 1:** Summary of selected DEGs in the testis of obese rats compared to the control.

Gene ID	Transcript ID	Gene Symbol	Description	Function	O/C FC
**Obesity and lipid metabolism**					
681337	NM_001109440	Acot4	acyl-CoA thioesterase 4	OM, fatty acid elongation, biosynthesis of unsaturated fatty acids	−10.75
24654	NM_001077641	Plcb1	phospholipase C beta 1	Protection against HFD-induced obesity, Aldosterone synthesis and secretion, regulation of acrosome reaction, positive regulation of fertilization, interleukin-12 production	−8.55
24254	NM_016997	Cel	carboxyl ester lipase	OM, Steroid biosynthesis, Fat digestion and absorption	−3.9
296138	NM_001106508	Acoxl	acyl-CoA oxidase-like	OM, fatty acid beta-oxidation	−4.72
690388	NM_001109587	Pla2g2f	phospholipase A2, group IIF	protection against HFD-induced obesity	3.0
24207	NM_001271053, NM_012501	Apoc3	apolipoprotein C3	OM, reduce obesity, negative regulation of lipid metabolism and transport, negative regulation of lipase activity	7.66
362540	NM_001108669	Hacd4	3-hydroxyacyl-CoA dehydratase 4	Fatty acid elongation, fatty acid metabolism	4.81
89813	NM_053551	Pdk4	pyruvate dehydrogenase kinase 4	OM, response to stress, cellular response to fatty acid, regulation of fatty acid oxidation, cellular response to stress	4.69
300438	NM_175762	Ldlr	Low-density lipoprotein receptor	Ovarian steroidogenesis, Aldosterone synthesis and secretion, Cortisol synthesis and secretion, cholesterol transport, intestinal cholesterol absorption, intestinal lipid	3.81
**Spermatogenesis, reproduction, fertilization**					
305938	NM_001191686	Spata13	spermatogenesis associated 13	Regulation of Rho and Ras signal transduction pathways, regulation of actin cytoskeleton	7.42
316426	NM_001014102	Spats2l	spermatogenesis associated, serine-rich 2-like	spermatogenesis	2.91
100362580	NM_001304424	Spag11bl	sperm associated antigen 11b-like	Sperm maturation	−3.18
360896	NM_203336	Esrrg	estrogen-related receptor gamma	spermatogenesis, regulation of transcription	−9.63
298961	NM_001106725	Agr2	anterior gradient 2, protein disulphide isomerase family member	Sperm maturation and fertilization, cell adhesion, response to stress	−7.8
**Cytochrome P450 (CYPs)**					
24303	NM_173093	Cyp2d3	Cytochrome P450, family 2, subfamily d, polypeptide 3	OM, Steroid hormone biosynthesis, Serotonergic synapse	−3.07
24894	NM_012692	Cyp2a1	Cytochrome P450, family 2, subfamily a, polypeptide 1	OM, Retinol metabolism, steroid hydroxylase activity, aromatase activity	2.51
56266	NM_019623	Cyp4f1	Cytochrome P450, family 4, subfamily f, polypeptide 1	OM, arachidonic acid metabolism, monooxygenase activity,	2.75
25429	NM_019138	Cyp7b1	Cytochrome P450, family 7, subfamily b, polypeptide 1	OM, Primary bile acid biosynthesis, localization of cell, monooxygenase activity,	3.49
25147	NM_017085	Cyp19a1	Cytochrome P450, family 19, subfamily a, polypeptide 1	aromatase	2.1
**Cell adhesion**					
364844	NM_001012215	Pcdhgb7	protocadherin gamma subfamily B, 7	OM	−2.96
81613	NM_001033860, NM_001033861, NM_001033862, NM_031755	Ceacam1	carcinoembryonic antigen-related cell adhesion molecule 1	protection from obesity	3.07
116711	NM_053919	Ceacam9	carcinoembryonic antigen-related cell adhesion molecule 9	protection from obesity	−2.74
**Immune response**					
288593	NM_001013045	Ccl24	C-C motif chemokine ligand 24	OM	3.57
362629	NM_001191869	Il22ra1	interleukin 22 receptor subunit alpha 1	OM, Cytokine-cytokine receptor interaction, Jak-STAT signaling pathway	5.53
24932	NM_012705	Cd4	Cd4 molecule	OM, Cytokine-cytokine receptor interaction	6.37
362417	NM_001004091	Il17re	interleukin 17 receptor E	OM	3.30
287910	NM_001004202	Ccl6	chemokine (C-C motif) ligand 6	OM	2.75
246759	NM_145672	Cxcl9	C-X-C motif chemokine ligand 9	OM	2.03
**Olfaction**					
405186	NM_001000886	Olr1710	olfactory receptor 1710	Olfactory transduction, perception of smell	−8.68
296694	NM_001000397	Olr434	olfactory receptor 434	OM, Olfactory transduction, perception of smell	−7.07
295743	NM_001000301	Olr472	olfactory receptor 472	OM, Olfactory transduction, perception of smell	−4.23

OM, obesity marker.

## Supplementary Materials

Supplementary materials can be found at https://www.mdpi.com/1422-0067/21/18/6493/s1. Table S1. Statistical summary of the obtained raw and trimmed data; Figure S1. Distribution of the detected genes with different number of zero count.
