# Supplementary material for: Transcriptome Analysis of Testis from HFD-Induced Obese Rats (Rattus norvigicus) Indicated Predisposition for Male Infertility
Source: Int J Mol Sci. 2020 Sep 5;21(18):6493. doi: 10.3390/ijms21186493 (PMC7554891; doi:10.3390/ijms21186493)
Supplement: Supplementary file 1 [file ijms-21-06493-s001.pdf]

Table S1. Statistical summary of the obtained raw and trimmed data.

No	Sample ID	Total read bases, 10^9	Total reads, 10^6	GC (%)	Q20 (%)	Q30 (%)	
1	C1	7.7	76.57	49.09	98.62	95.85	Raw
2	C2	6.5	64.41	48.50	98.57	95.73	
3	C3	7.2	68.53	48.61	98.63	95.41	
4	O1	6.9	68.69	48.75	98.53	95.73	
5	O2	8.1	80.27	49.96	98.89	96.37	
6	O3	7.6	77.32	49.05	98.72	96.32	
1	C1	7.6	75.67	49.10	99.01	96.41	Trimmed
2	C2	6.4	63.66	48.52	98.96	96.30	
3	C3	7.1	67.12	48.72	98.76	96.51	
4	O1	6.8	67.73	48.77	99.00	96.38	
5	O2	8.0	79.64	49.97	99.17	96.77	
6	O3	7.5	67.48	49.39	99.21	96.68	

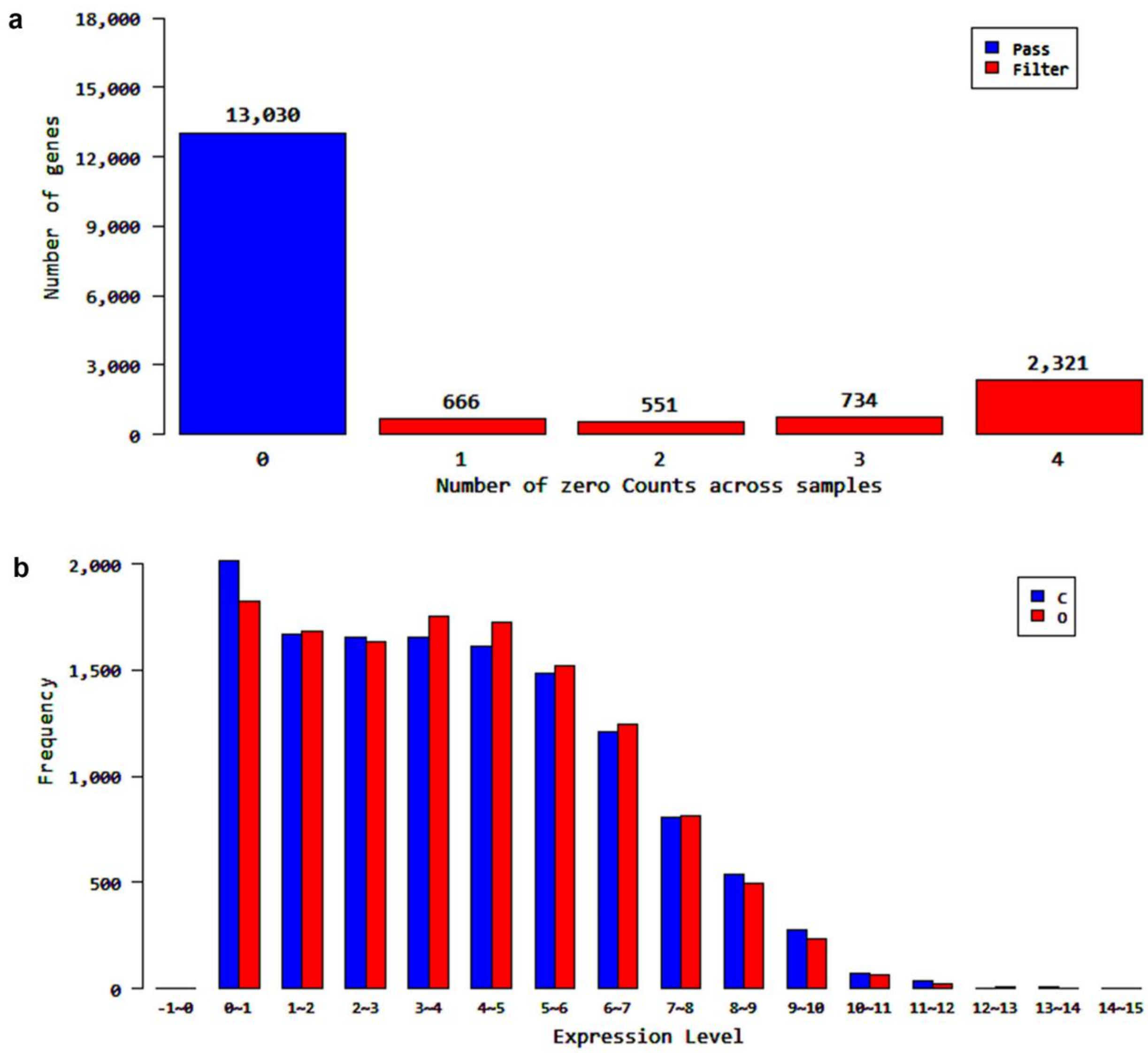

Figure S1. Distribution of the detected genes with different number of zero count. Zero count indicates that the gene was not detected in at least one sequencing read. b. Distribution of gene expression level in testis of obese rats (red) compared to the control (blue).
